# Macular pucker formation after inverted internal limiting membrane flap technique: Two case reports

**DOI:** 10.1016/j.ajoc.2022.101282

**Published:** 2022-01-20

**Authors:** Keisuke Kanda, Hiroshi Nakashima, Kazuyuki Emi

**Affiliations:** From the Department of Ophthalmology, Osaka Rosai Hospital Clinical Research Center for Optical Sensory Organ Disability, Osaka, Japan

**Keywords:** Macular hole, Inverted flap technique, Postoperative complications, Internal limiting membrane, Vitrectomy, Histopathological examination

## Abstract

**Purpose:**

The inverted internal limiting membrane (ILM) flap technique is generally used to treat refractory macular holes (MHs). Recently, a case of macular pucker formation outside the ILM flap after using silicone oil was reported. Although the pucker formation was attributed to the silicone oil use in that case, here we report two cases of macular pucker that occurred after the inverted ILM flap technique was performed without silicone oil. In one case, the ILM flap and proliferated tissue was removed, followed by their histopathological examination.

**Observations:**

Two patients with MH underwent vitrectomies using the inverted ILM flap technique. In both patients, the visual acuity worsened postoperatively, and macular pucker formation, associated with the ILM flap, was observed. In one patient, visual acuity improved after ILM flap removal, and histopathological examination of the specimen indicated strong cellular proliferation between the ILMs.

**Conclusions and Importance:**

Following the inverted ILM flap technique, macular pucker may occur even without the use of silicone oil. Removal of the flap and associated proliferative tissue was effective and resulted in no recurrence of MH or pucker. Ophthalmologists should consider the possibility that tissues on the ILM may lead to macular pucker formation especially inside the flap, in the area between the ILMs.

## 1 Introduction

Macular hole (MH) surgery was originally reported by Kelly and Wendel,^1^ and has evolved considerably over the past decades. The inverted internal limiting membrane (ILM) flap technique was subsequently developed, and this method is considered to improve the MH closure rate.^2^ Remnant ILM on the macula following the inverted ILM flap technique was reported to have no effect on final visual acuity.^3^ However, a recent study reported on the postoperative complications associated with the ILM flap technique.^4^ In that study, macular pucker was formed outside the ILM flap, and the silicone oil used in the procedure was considered to have a significant role in the formation of proliferative tissue. Here, we report two cases of macular pucker that occurred after the inverted ILM flap technique even without the use of silicone oil. In one case, the visual acuity improved after removal of the flap and associated proliferative tissue, and subsequent histopathological examination showed strong proliferation between ILMs. Tissues on the ILM may lead to macular pucker formation inside the flap, especially in the area between the ILMs.

## 2 Findings

We reviewed the medical records of 26 patients at Osaka Rosai Hospital who underwent the inverted flap technique between 2016 and 2020. Two patients had postoperative disruption of the retinal layer structure at the macular to the extent that they complained of vision loss and distortion. In this report, we describe the clinical course of these two patients.

### 2.1 Case 1

**Fig. 1:**
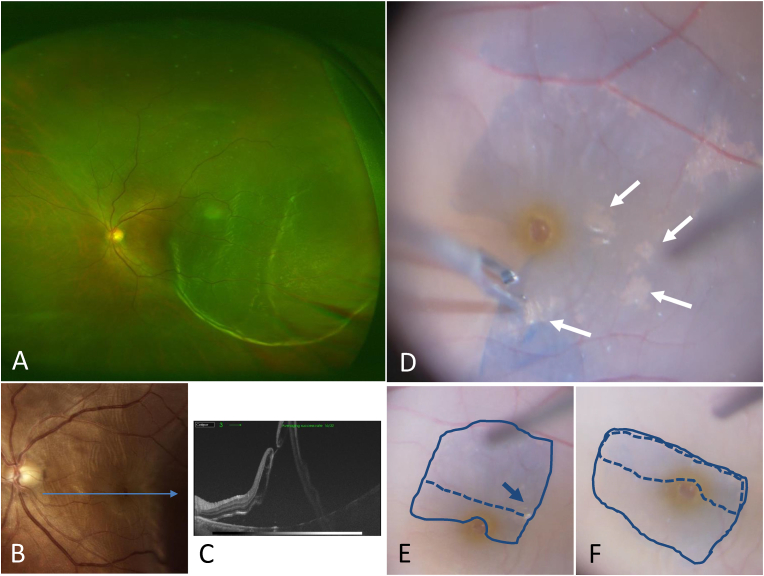
Case 1. Preoperative and intraoperative fundus and optical coherence tomography (OCT) findings. A, B: Fundus photographs acquired at the first visit. Retinal detachment involving the superior and temporal retina (A) with fovea-off is observed (B). C: Horizontal swept-source OCT findings for the first visit showss macular hole and no adhesion between the vitreous and the macular (C). D, E, F: Intraoperative images obtained during the inverted internal limiting membrane (ILM) flap technique. There were hyperreflective areas (white arrows) on the vitreous side of the ILM before the inversion that were slightly difficult to stain with Brilliant Blue G (BBG) (D). The ILM was peeled, except the area of the inverted flap, and it was gently flipped at the fracture broken line. The arrow shows hyperreflective area on ILM (E). The ILM flap was flipped to cover the entire MH and overlaps in the part surrounded by the broken line. Therefore, the hyperreflective area was consequently sandwiched between the ILMs (F). (For interpretation of the references to colour in this figure legend, the reader is referred to the Web version of this article.)

**Fig. 2:**
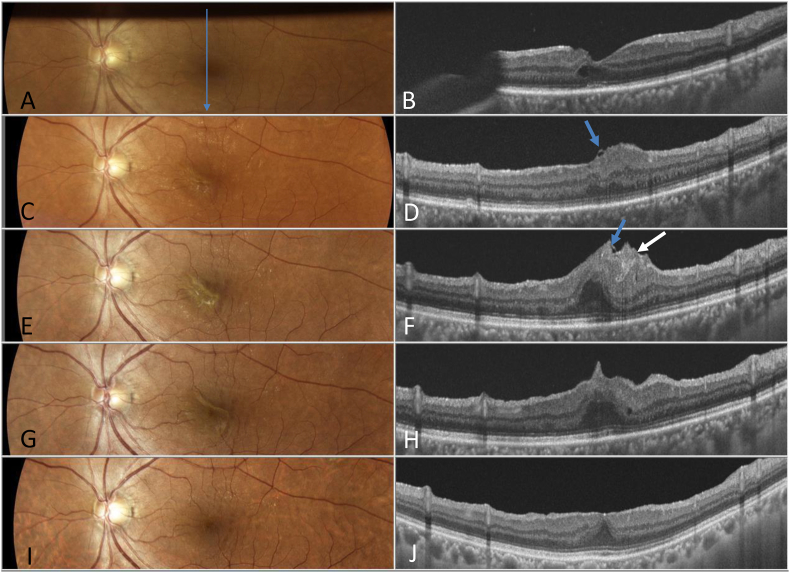
Case 1. Fundus and vertical optical coherence tomography (OCT) findings after the first and second surgery. A, B: Images acquired one week after the first surgery showing the shadow of the gas line. The retina was reattached (A), and macular hole (MH) was closed (B). C, D: One month after the first surgery. A slight macular pucker is observed (C). Absence of foveal depression and disturbance of retinal layer structure are observed on OCT. A part of the inverted internal limiting membrane (ILM) flap (blue arrow) is observed (D). E, F: 3 months after the first surgery. The pucker ridge appears steep (E). OCT image showing a localized, highly reflective area (white arrow) under the inverted ILM (blue arrow) (F). G, H: The day after the second operation. The pucker was removed, following which the macular appears flatter (G). The localized tissue proliferation was removed, and the retina was centrifugally relaxed (H). I, J: One year after the reoperation. The macular appears flat, indicating its return to normalcy (I). No recurrence of MH or macular pucker is observed with OCT (J). (For interpretation of the references to colour in this figure legend, the reader is referred to the Web version of this article.)

**Fig. 3:**
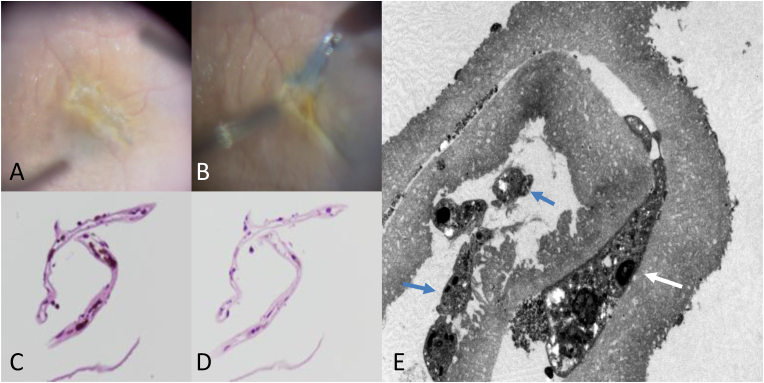
Case 1. Intraoperative findings during second operation, and histological findings for the removed membrane. A, B: Intraoperative view from the perspective of the surgeon. The macular pucker showing ridge formation (A). The proliferative internal limiting membrane (ILM) flap is adhered firmly to the retina. It was possible to detach it as a single piece using an end-gripping forceps (B). C, D, E: Histological examination of the removed ILM flap by light and electron microscopy (LM, EM). LM shows cell proliferation is observed predominantly and adhesively in the area sandwiched between the ILMs. These proliferative cells contain melanin pigment, as detected by hematoxylin and eosin staining (H & E stain) (C). The melanin-rich cells are bleached using potassium permanganate (KMnO_4_) and oxalic acid (D). The vitreous side of the ILM is smooth and continuous while the retinal side is characterized by irregular undulations under EM. Proliferative cells are adhering between the vitreous side of the ILMs (white arrow). The cells found outside the ILM flap (blue arrows) do not have strong adhesion to the retinal side of the ILM (E). (For interpretation of the references to colour in this figure legend, the reader is referred to the Web version of this article.)

A 59-year-old male patient with at least a 10-day history of visual disturbance and visual field defect in his left eye was referred to Osaka Rosai Hospital. Best-corrected visual acuity (BCVA) at presentation was 0.2. Moderate cataract, and retinal detachment involving the superior and temporal retina with fovea-off were observed (Fig. 1A and B). Optical coherence tomography (OCT) showed MH and no adhesion between the vitreous to the macula (Fig. 1C). The surgeon decided to perform phacovitrectomy since the patient had MH. After obtaining informed consent from the patient, a combined vitrectomy with phacoemulsification and intraocular lens implantation was performed on the day of the first visit. The small tears in the upper quadrant associated with the posterior vitreous detachment (PVD) were the cause of the retinal detachment. There was no adhesion between the vitreous and the macula. After performing core vitrectomy, the surgeon extended the PVD and performed peripheral vitreous shaving with scleral indentation. The subretinal fluid was too viscous to drain completely. The surgeon performed the inverted flap technique to assist the MH closure in case the residual subretinal fluid would accumulate at the post pole postoperatively. To identify the ILM, a mixture of Brilliant Blue G (BBG) and sodium hyaluronate was used (Opegan; Santen Pharmaceuticals Co, Ltd, Osaka, Japan).^5^ There were hyperreflective areas on the vitreous side of the ILM before the inversion that were slightly difficult to stain with BBG (D). The ILM was peeled away except at the area of the flap. The flap had a hyperreflective area before it was inverted (Fig. 1E). The ILM flap was gently flipped to cover the entire MH from its superior margin, and the hyperreflective area was consequently sandwiched between the ILMs (Fig. 1F). Viscoelastic (Shellgan; Santen Pharmaceuticals Co, Ltd, Osaka, Japan) was placed over the ILM flap. After fluid-air exchange was performed, maximal subretinal fluid was drained from a drainage hole intentionally placed at the temporal retina. Diathermic coagulation and laser photocoagulation were then performed around the retinal breaks and the drainage hole. The intraocular tamponade agent used was 20% sulfur hexafluoride (SF_6_). We instructed the patient to assume a prone position postoperatively. One week postoperatively, the retina was reattached (Fig. 2A), and follow-up OCT showed complete MH closure (Fig. 2B). At one month postoperatively, the patient's BCVA improved to 0.4. Nevertheless, slight macular pucker was observed (Fig. 2C). OCT showed absence of foveal depression and disturbance of the retinal layer structure (Fig. 2D). At 3 months postoperatively, the patient complained of distortion. The pucker ridge had become steep (Fig. 2E). The foveal deformation worsened, and OCT showed tissue proliferation confined to a highly reflective area under the inverted ILM (Fig. 2F). The ILM flap and proliferated cells were peeled off 4 months after the first operation, and retinal distortion improved thereafter (Fig. 2G–J). Although the cell proliferation on the ILM flap caused it to adhere firmly to the retina, it could still be detached as a single piece using an end-gripping forceps (Fig. 3A and B). The day after the second operation, the pucker had completely resolved. The retina appeared flatter than before in fundus photography (Fig. 2G), and was centrifugally relaxed in OCT (Fig. 2H). The removed ILM flap was divided into two pieces. One piece was immediately immersed into 10% neutral-buffered formalin, and the other into 2.5% buffered glutaraldehyde. These specimens were processed for light and electron microscopy (LM and EM), respectively. In the LM, cell proliferation was observed predominantly and adhesively in the area sandwiched between the ILMs (Fig. 3C). These cells contained melanin pigment based on hematoxylin and eosin staining (H & E stain), and the cells were adhered to each other to form a macular pucker between the ILMs. We performed a melanin bleaching procedure using potassium permanganate (KMnO_4_) and oxalic acid to confirm the presence of melanin pigment in these proliferating cells. The melanin-rich cells were bleached with this method (Fig. 3D) and were considered retinal pigment epithelium (RPE) cells. With EM, the proliferated cells were found strongly adhered to the vitreous side of the ILM (Fig. 3E), though the cells outside the ILM flap were not. After the second operation, the foveal structure and patient's complaint of distortion improved. BCVA improved to 1.2 one year after the second operation. No recurrence of macular pucker has been observed since (Fig. 2I and J).

### 2.2 Case 2

**Fig. 4:**
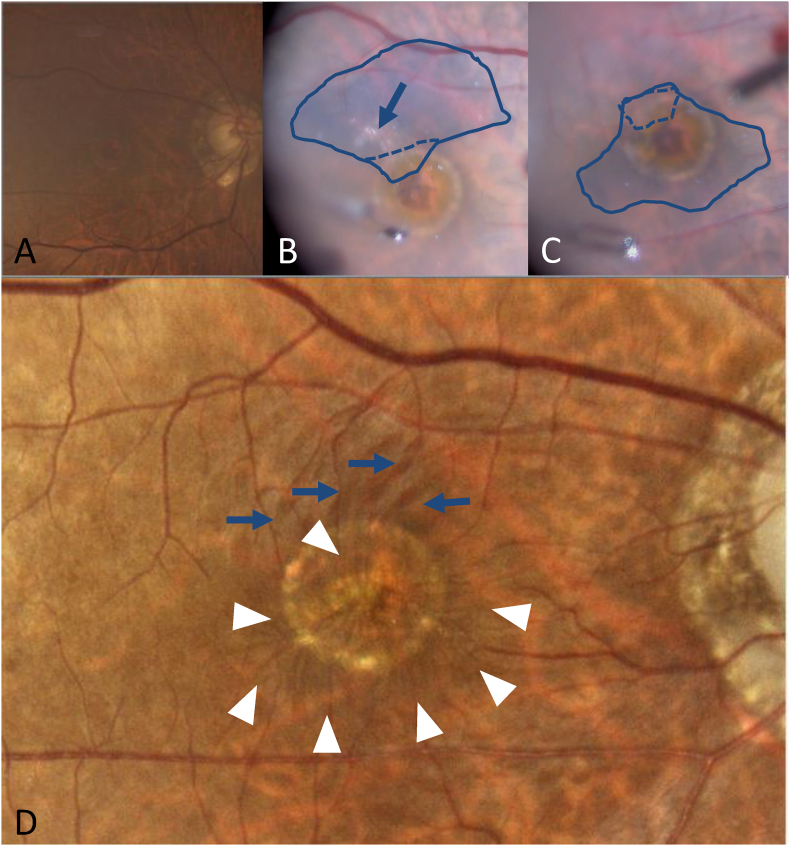
Case 2. Preoperative, intraoperative, and postoperative fundus images. A: Fundus photograph acquired at the first visit. A macular hole (MH) is observed, although it is unclear due to cataract (A). B, C: Intraoperative findings during inverted internal limiting membrane (ILM) flap technique. The ILM was peeled away, except the area for an inverted flap, and it was gently flipped at the broken line. The arrow shows a hyperreflective area on the ILM that was slightly difficult to stain with Brilliant Blue G (BBG) (B). The ILM flap was flipped to cover the whole MH and overlaps in the part surrounded by the broken line. Therefore, the hyperreflective area was consequently sandwiched between the ILMs (C). D: Fundus photograph acquired 3 months after the operation. Macular pucker is found in the area surrounded by white arrow heads. The blue arrow indicates a dissociated optic nerve fiber layer (D). (For interpretation of the references to colour in this figure legend, the reader is referred to the Web version of this article.)

**Fig. 5:**
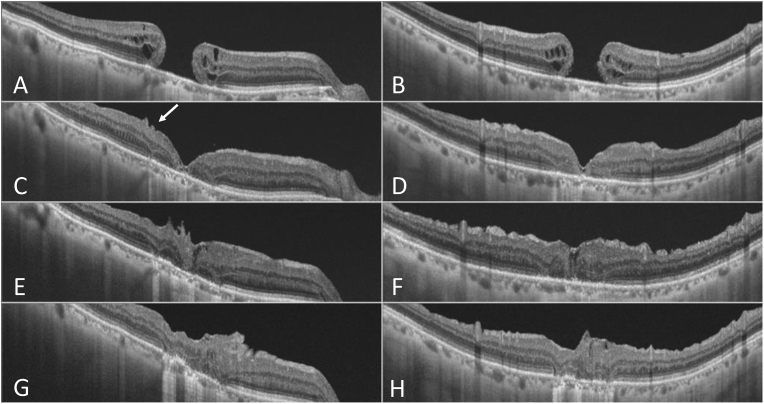
Case 2. Preoperative and postoperative optical coherence tomography (OCT) findings. A, C, E, G: Horizontal images. B, D, F, H: Vertical images. Full-thickness macular hole (MH) with complete posterior vitreous detachment (PVD) is observed at the first visit (A, B). MH closure is observed one month postoperatively. Slight wrinkles (white arrow) can be noted on the temporal side of the fovea (C, D). Three months after the operation, the wrinkles worsened. Absence of foveal depression and disturbance of the retinal layer structure are observed (E, F). One year after the operation, the retinal structure shows no improvement (G, H).

A 72-year-old male patient was referred to our department after experiencing about 6 months of decreased visual acuity in his right eye secondary to a chronic MH. At presentation, his BCVA was 0.2, and MH and moderate cataract were observed (Fig. 4A). OCT showed full-thickness MH with complete PVD. The minimum size of the MH was 748 μm. There were no retinal tears. A surgery was performed using the inverted ILM flap technique, similar to that described in Case 1, at one month after the first visit. There was a hyperreflective area that appeared on the ILM intraoperatively that was slightly difficult to stain with BBG (Fig. 4B). The ILM flap was inverted to cover the entire MH, and the hyperreflective area was consequently sandwiched between the ILMs (Fig. 4C). For tamponade, the vitreous cavity was filled with air. One month after the surgery, the patient's visual acuity improved to 0.7. However, at 3 months postoperatively, the patient complained of vision loss and distortion, and his BCVA had decreased to 0.2. Fundus photographs showed the occurrence of pucker (Fig. 4D). Fig. 5 shows the preoperative (Fig. 5A and B) and postoperative (Fig. 5C–H) changes in OCT. At one month postoperatively, although MH closure was observed with OCT, there were slight wrinkles on the temporal side of the fovea (Fig. 5C and D). Three months after surgery, OCT showed no foveal depression or disturbance of the retinal layer structure (Fig. 5E and F). The patient did not wish to have the ILM flap and proliferated cells removed by a second operation. Although the MH had not recurred, the foveal deformation has not improved and the patient's BCVA decreased to 0.1 one year postoperatively (Fig. 5G and H).

## 3 Discussion

In this report, we describe two cases of macular pucker formation that developed after the inverted ILM flap technique. Visual acuity worsened in both cases after the initial surgery. In one case, the macular pucker was removed, and histopathological examination of the surgical specimen showed proliferation of RPE cells and their encapsulation in the ILM flap. In particular, proliferation was mainly observed on the vitreous side of the ILM, which caused strong adhesion between the ILMs. Visual acuity was well maintained after a second operation. Removal of the ILM flap was done safely without bleeding or excessive traction that would impair macular function. There was no recurrence of MH or macular pucker.

Our report is novel because the cell proliferation occurred between the ILMs even without the use of silicone oil during the ILM flap technique. There is only one other report of postoperative complication associated with the ILM flap technique.^4^ In that report, macular pucker occurred after the inverted ILM flap technique was performed with silicone oil for MH repair. In that case, it was assumed that the postoperative inflammation caused by the silicone oil had a significant role in inducing tissue proliferation on the surface of the ILM flap, that was, the originally retinal side of the ILM. The report did not show proliferation on the vitreous side of the ILM or inside the ILM flap. In contrast, in Case 1 reported herein, the proliferation had formed between ILMs even without the use of silicone oil, suggesting that substances adhering on the vitreous side of the ILM may have caused the proliferation.

There might be an indeterminate quantity of cells and collagen remaining on the vitreous side of the ILM, which serve as a scaffold for cell proliferation.^6^^,^^7^ Although the epiretinal membrane was not obvious before the surgery in our cases, there were hyperreflective areas on the vitreous side of the ILM flap before the inversion that were slightly difficult to stain with BBG. Another study of cases showing patchy, incomplete staining before ILM peeling in MH surgery showed that there was significantly more vitreous surface tissue on the ILM in patients with an incomplete staining pattern than in those with a uniform staining pattern.^8^ It is possible that there were residual cells and collagen in the hyperreflective areas in our cases. Moreover, the inverted ILM flap itself has been reported to function as a scaffold for the proliferation and migration of Müller cells during MH closure.^9^ In such an environment where cellular proliferation is active, accumulation of cell remnants and collagen on the vitreous side of the ILM could lead to macular pucker following inverted flap technique in some cases. Therefore, it is necessary to consider that the tissues that form on the ILM may lead to macular pucker formation following an inverted ILM flap technique. In addition, based on the pathological findings, we consider that the entrapment of cells between the ILMs could have further enhanced the proliferation. The inverted flap technique applied with a smaller overlapping area of the ILM could prevent the enhancement of cell proliferation inside the flap.

It should be noted that the mechanism of proliferation following the inverted flap technique in MH with rhegmatogenous retinal detachment (RRD) could be different from that in MH without RRD. The presence of RPE cells in the histological section strongly suggests that the RPE cells distributed through the retinal tears preoperatively or postoperatively could have been involved in the proliferation.^10^ Although histopathological examination was not performed in case 2, the cells involved in proliferation following the inverted flap technique could differ depending on the existence of retinal RRD, and the clinical characteristics may also differ.

Although the number of cases in this report is limited, and one case was accompanied by retinal detachment, we have found that macular pucker formed in 2 of 26 cases in which the inverted ILM flap technique was performed at our hospital. Hence, cell proliferation on the ILM flap can occur much more frequently than expected. In any case, frequent follow-up examinations are preferable after using the inverted flap technique, since early surgical intervention may improve visual recovery, as reported in Case 1.

## 4 Conclusion

We experienced two cases of decreased visual acuity due to macular pucker formation that occurred following the inverted flap technique. In one case, removal of the ILM flap improved visual acuity and protected against future recurrence of MH or pucker. Histopathological examination of the surgical specimens indicated strong proliferation between the ILMs inside the flap. It is necessary to consider that the tissues that form on the ILM may lead to macular pucker formation following an inverted ILM flap technique. Further studies are needed to clarify the characteristics of cellular proliferation associated with the ILM flap and the prognosis after its removal by a second operation.

## Patient consent

Written informed consent was obtained from the patients for publication of this case report and any accompanying images.

## Funding

No funding or grant support.

## Authorship

All authors attest that they meet the current ICMJE criteria for authorship.

## Financial disclosures

K.E. has received lecture fees form Alcon, Hoya, Kowa, Novartis, Otsuka, and Santen.
